# Complete genome sequence of *Geobacillus thermoglucosidasius* C56-YS93, a novel biomass degrader isolated from obsidian hot spring in Yellowstone National Park

**DOI:** 10.1186/s40793-015-0031-z

**Published:** 2015-10-05

**Authors:** Phillip J. Brumm, Miriam L. Land, David A. Mead

**Affiliations:** Lucigen Corporation, Middleton, WI USA; Oak Ridge National Laboratory, Oak Ridge, TN USA; Great Lakes Bioenergy Research Center, University of Wisconsin, Madison, WI USA

**Keywords:** *Geobacillus thermoglucosidasius* C56-YS93, Hot springs, Biomass, Xylan, Prophage

## Abstract

*Geobacillus thermoglucosidasius* C56-YS93 was one of several thermophilic organisms isolated from Obsidian Hot Spring, Yellowstone National Park, Montana, USA under permit from the National Park Service. Comparison of 16 S rRNA sequences confirmed the classification of the strain as a *G. thermoglucosidasius* species. The genome was sequenced, assembled, and annotated by the DOE Joint Genome Institute and deposited at the NCBI in December 2011 (CP002835). The genome of *G. thermoglucosidasius* C56-YS93 consists of one circular chromosome of 3,893,306 bp and two circular plasmids of 80,849 and 19,638 bp and an average G + C content of 43.93 %. *G. thermoglucosidasius* C56-YS93 possesses a xylan degradation cluster not found in the other *G. thermoglucosidasius* sequenced strains. This cluster appears to be related to the xylan degradation cluster found in *G. stearothermophilus. G. thermoglucosidasius* C56-YS93 possesses two plasmids not found in the other two strains. One plasmid contains a novel gene cluster coding for proteins involved in proline degradation and metabolism, the other contains a collection of mostly hypothetical proteins.

**Electronic supplementary material:**

The online version of this article (doi:10.1186/s40793-015-0031-z) contains supplementary material, which is available to authorized users.

## Introduction

Identification of new organisms that produce biomass-degrading enzymes is of considerable interest. Commercial uses for these enzymes include paper manufacturing, brewing, biomass deconstruction and the production of animal feeds [1–3]. Hot springs, especially those at Yellowstone National Park, have been a source of many new organisms including *Thermus aquaticus* [4, 5], *Thermus brockianus* [6], and *Acidothermus cellulolyticus* [7] that possess enzymes with significant potential in biotechnological applications [8]. As part of a project in conjunction with the Great Lakes Bioenergy Research Center, Dept. of Energy, C5-6 Technologies and Lucigen Corp. isolated, characterized, and sequenced a number of new enzyme-producing aerobic organisms from Yellowstone hot springs.

*Geobacillus* species were the most common aerobic organisms isolated during the cultivation of most hot springs samples. *Geobacillus* species were originally classified as members of the genus *Bacillus*, but were reclassified as a separate genus based on 16S rRNA gene sequence analysis, lipid and fatty acid analysis, phenotypic characterization, and DNA–DNA hybridization experiments [9]. *Geobacillus* species have been isolated from a number of hostile environments including high-temperature oilfields [10], a corroded pipeline in an extremely deep well [11], African [12] and Russian [13] hot springs, marine vents [14], and the Mariana Trench [15], yet they can also be found in garden soils [16] and hay composts [17]. In many cases though, it is unclear if these isolations of *Geobacillus* species represent vegetative cells growing in these environments or merely spores spread from other locations [18]. The ability of *Geobacillus* species to thrive in varied and often hostile environments suggests that these species possess enzymes suitable for applications in hostile industrial environments. We therefor sequenced a number of these *Geobacillus* isolates including *G. thermoglucosidasius* C56-YS93 to identify new enzymes suitable for use in biomass conversion into fuels and chemicals.

### Organism Information

#### Classification and Features

**Fig. 1:**
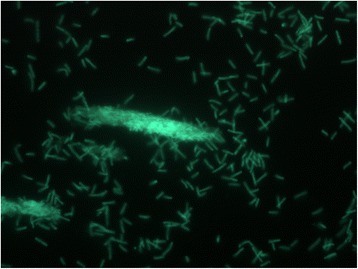
Micrograph of *Geobacillus thermoglucosidasius* C56-YS93 cells showing individual cells and clumps of cells. Cells were grown in TSB plus 0.4 % glucose for 18 h. at 70 °C. A 1.0 ml aliquot was removed, centrifuged, re-suspended in 0.2 ml of sterile water, and stained using a 50 μM solution of SYTO® 9 fluorescent stain in sterile water (Molecular Probes). Dark field fluorescence microscopy was performed using a Nikon Eclipse TE2000-S epifluorescence microscope at 2000× magnification using a high-pressure Hg light source and a 500 nm emission filter

*G. thermoglucosidasius* C56-YS93 is one of a number of novel thermophilic species isolated from Obsidian Hot Spring, Yellowstone National Park, Montana, USA (44.6100594° latitude and −110.4388217° longitude) under a sampling permit from the National Park Service. The hot spring possesses a pH of 6.37 and a temperature range of 42–90 °C. The organism was isolated from a sample of hot spring water by enrichment and plating on YTP-2 medium (YTP-2 media contains (per liter) 2.0 g yeast extract, 2.0 g tryptone, 2.0 g sodium pyruvate, 1.0 g KCl, 2.0 g KNO_3_, 2.0 g Na_2_HPO_4_.7H_2_O, 0.1 g MgSO_4_, 0.03 g CaCl_2_, and 2.0 ml clarified tomato juice) at 70 °C. The culture is freely available from the *Bacillus* Genetic Stock Center (BGSC). Cultures are routinely grown on tryptic soy broth without glucose (TSB) (Difco) media and maintained on TSB agar plates. C5-6 Technologies, Lucigen, the National Park Service, and the Joint Genome Institute have placed no restrictions on the use of the culture or sequence data. *G. thermoglucosidasius* C56-YS93, is a gram-positive, rod-shaped facultative anaerobe, (Table 1, Additional file 1: Table S1), with optimum growth temperature of 65 °C and maximum growth temperature of 75 °C. This is similar to the optimum growth temperature reported for *G. thermoglucosidasius* TNO-09.020 [19], but significantly higher than reported for previously isolated strains (<60 °C) [9]. *G. thermoglucosidasius* C56-YS93 appears to grow as a mixture of single cells and large clumps in liquid culture (Fig. 1).

**Table 1:** Classification and general features of *Geobacillus thermoglucosidasius* C56-YS93 [48]

MIGS ID	Property	Term	Evidence code^a^
	Classification	Domain *Bacteria*	TAS [49]
		Phylum *Firmicutes*	TAS [9]
		Class *Bacilli*	TAS [9]
		Order *Bacillales*	TAS [9]
		Family *Bacillaceae*	TAS [9]
		Genus *Geobacillus*	TAS [9]
		Species *Geobacillus thermoglucosidasius*	TAS [9]
		Strain C56-YS93	
	Gram stain	Positive	IDA
	Cell shape	Rods and chains of rods	IDA
	Motility	Motile	IDA
	Sporulation	Subterminal spores	IDA
	Temperature range	55 to 75 °C	IDA
	Optimum temperature	65 °C	IDA
	pH range; Optimum	5.8–8.0; 7.5	IDA
	Carbon source	Carbohydrate or protein	IDA
GS-6	Habitat	Hot spring	
MIGS-6.3	Salinity	Not reported	IDA
MIGS-22	Oxygen requirement	Facultative anaerobe	IDA
MIGS-15	Biotic relationship	Free-living	IDA
MIGS-14	Pathogenicity	Non-pathogen	IDA
MIGS-4	Geographic location	Obsidian Spring, Yellowstone National Park*,* USA	IDA
MIGS-5	Sample collection	September 2003	IDA
MIGS-4.1	Latitude	44.6603028	TAS [50]
MIGS-4.2	Longitude	−110.865194	TAS [50]
MIGS-4.4	Altitude	2416 m	TAS [50]

^a^Evidence codes - *IDA* inferred from direct assay, *TAS* traceable author statement (i.e., a direct report exists in the literature), *NAS* non-traceable author statement (i.e., not directly observed for the living, isolated sample, but based on a generally accepted property for the species, or anecdotal evidence). These evidence codes are from http://www.geneontology.org/GO.evidence.shtml of the Gene Ontology project [51]

**Fig. 2:**
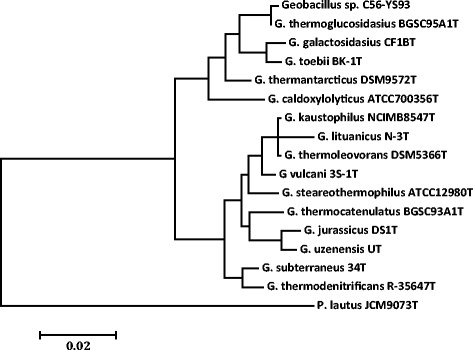
Molecular phylogenetic analysis by Maximum Likelihood method as detailed in the Material and Methods section. The tree with the highest log likelihood (−3014.19) is shown. The tree is drawn to scale, with branch lengths measured in the number of substitutions per site. The novel sequenced *Geobacillus* strains are indicated in bold. The type strains of all validly described species are included (NCBI accession numbers): *G. caldoxylolyticus* ATCC700356^T^ (AF067651), *G. galactosidasius* CF1B^T^ (AM408559), *G. jurassicus* DS1^T^ (FN428697), *G. kaustophilus* NCIMB8547T (X60618), *G. lituanicus* N-3^T^ (AY044055), *G. stearothermophilus* R-35646^T^ (FN428694), *G. subterraneus* 34 T (AF276306), *G. thermantarcticus* DSM9572T(FR749957), *G. thermocatenulatus* BGSC93A1^T^ (AY608935), *G. thermodenitrificans* R-35647^T^ (FN538993), G. thermoglucosidasius BGSC95A1^T^ (FN428685), *G. thermoleovorans*DSM5366^T^ (Z26923), *G. toebii* BK-1^T^ (FN428690), *G. uzenensis* U^T^ (AF276304) and *G. vulcani* 3S-1^T^ (AJ293805). The 16S rRNA sequence of *Paenibacillus lautus*JCM9073^T^ (AB073188) was used to root the tree

A phylogenetic tree was constructed to identify the relationship of *G. thermoglucosidasius* C56-YS93 to other members of the *Geobacillus* family. The phylogeny of *G. thermoglucosidasius* C56-YS93 was determined using its 16S rRNA gene sequence, as well as those of the type strains of all validly described *Geobacillus* spp. The 16S rRNA gene sequences were aligned using MUSCLE [20], pairwise distances were estimated using the Maximum Composite Likelihood (MCL) approach, and initial trees for heuristic search were obtained automatically by applying the Neighbour-Joining method in MEGA 5 [21]. The alignment and heuristic trees were then used to infer the phylogeny using the Maximum Likelihood method based on Tamura-Nei [22]. The phylogenetic tree confirms the identification of *G. thermoglucosidasius* C56-YS93 as a *G. thermoglucosidasius* sp. (Fig. 2).

### Genome sequencing information

#### Genome project history

*G. thermoglucosidasius* C56-YS93 was selected for sequencing on the basis of its biotechnological potential as part of the U.S. Department of Energy’s Genomic Science program (formerly Genomics:GTL). The genome sequence is deposited in the Genomes On Line Database [23, 24] (GOLD ID = Gc01858), and in GenBank (NCBI Reference Sequence = CP002835.1). Sequencing, finishing and annotation were performed by the DOE Joint Genome Institute (JGI). A summary of the project information and its association with MIGS identifiers is shown in Table 2.

**Table 2:** Project information

MIGS ID	Property	Term
MIGS 31	Finishing quality	Finished
MIGS 28	Libraries used	6 and 24 kb
MIGS 29	Sequencing platforms	454 Titanium, Illumina GAii
MIGS 31.2	Fold coverage	5.8
MIGS 30	Assemblers	Phred/Phrap/Consed
MIGS 32	Gene calling method	Prodigal, GenePRIMP
	Locus tag	GEOTH
	Genbank ID	CP002835.1
	Genbank date of release	Dec. 1, 2011
	GOLD ID	Gc01858
	BIOPROJECT	PRJNA40781
MIGS 13	Project relevance	Biotechnological
	Source material identifier	Genome

### Growth conditions and genomic DNA preparation

For preparation of genomic DNA, 1 l cultures of *G. thermoglucosidasius* C56-YS93 were grown from a single colony in YTP-2 medium at 70 °C in flasks agitated at 200 rpm and collected by centrifugation. Culture stocks were maintained on YTP-2 agar plates grown at 70 °C. The cell concentrate was lysed using a combination of SDS and proteinase K, and genomic DNA was isolated using a phenol/chloroform extraction [25]. The genomic DNA was precipitated, and treated with RNase to remove residual contaminating RNA.

### Genome sequencing and assembly

The genome of *G. thermoglucosidasius* C56-YS93 was sequenced at the JGI using a combination of Illumina and 454 technologies [26]. An Illumina GAii shotgun library with reads of 878 Mb, a 454 Titanium draft library with average read length of 510–525 bp bases, and a paired end 454 library with average insert size of 13 Kb were generated for this genome. All general aspects of library construction and sequencing performed at the JGI [27]. Illumina sequencing data was assembled with VELVET [28], and the consensus sequences were shredded into 1.5 kb overlapped fake reads and assembled together with the 454 data. Draft assemblies were based on 197.18 MB 454 draft data, and all of the 454 paired end data. Newbler parameters are consed -a 50–1 350 –g –m –ml 20. The initial Newbler assembly contained 54 contigs in 2 scaffolds. We converted the initial 454 assembly into a phrap assembly by making fake reads from the consensus and collecting the read pairs in the 454 paired end library. The Phred/Phrap/Consed software package was used for sequence assembly and quality assessment ([29–31] in the following finishing process. Illumina data was used to correct potential base errors and increase consensus quality using a software Polisher developed at JGI (Alla Lapidus, unpublished). After the shotgun stage, reads were assembled with parallel phrap (High Performance Software, LLC). Possible mis-assemblies were corrected with gapResolution (Cliff Han, unpublished), Dupfinisher (Han, 2006), or sequencing cloned bridging PCR fragments with subcloning. Gaps between contigs were closed by editing in Consed, by PCR and by Bubble PCR primer walks. A total of 301 additional reactions and 7 shatter libraries were necessary to close gaps and to raise the quality of the finished sequence. The total number of reads used in final assembly was 190,696. The overall average error rate of the final assembly was 0.02 errors/10 kb.

### Genome annotation

Genes were identified using Prodigal [32] as part of the Oak Ridge National Laboratory genome annotation pipeline, followed by a round of manual curation using the JGI GenePRIMP pipeline [33]. The predicted CDSs were translated and used to search the National Center for Biotechnology Information (NCBI) nonredundant database, UniProt, TIGRFam, Pfam, PRIAM, KEGG, COG, and InterPro databases. These data sources were combined to assert a product description for each predicted protein. Non-coding genes and miscellaneous features were predicted using tRNAscan-SE [33], RNAMMer [34], Rfam [35], TMHMM [36], and signalP [36].

### Genome properties

**Fig. 3:**
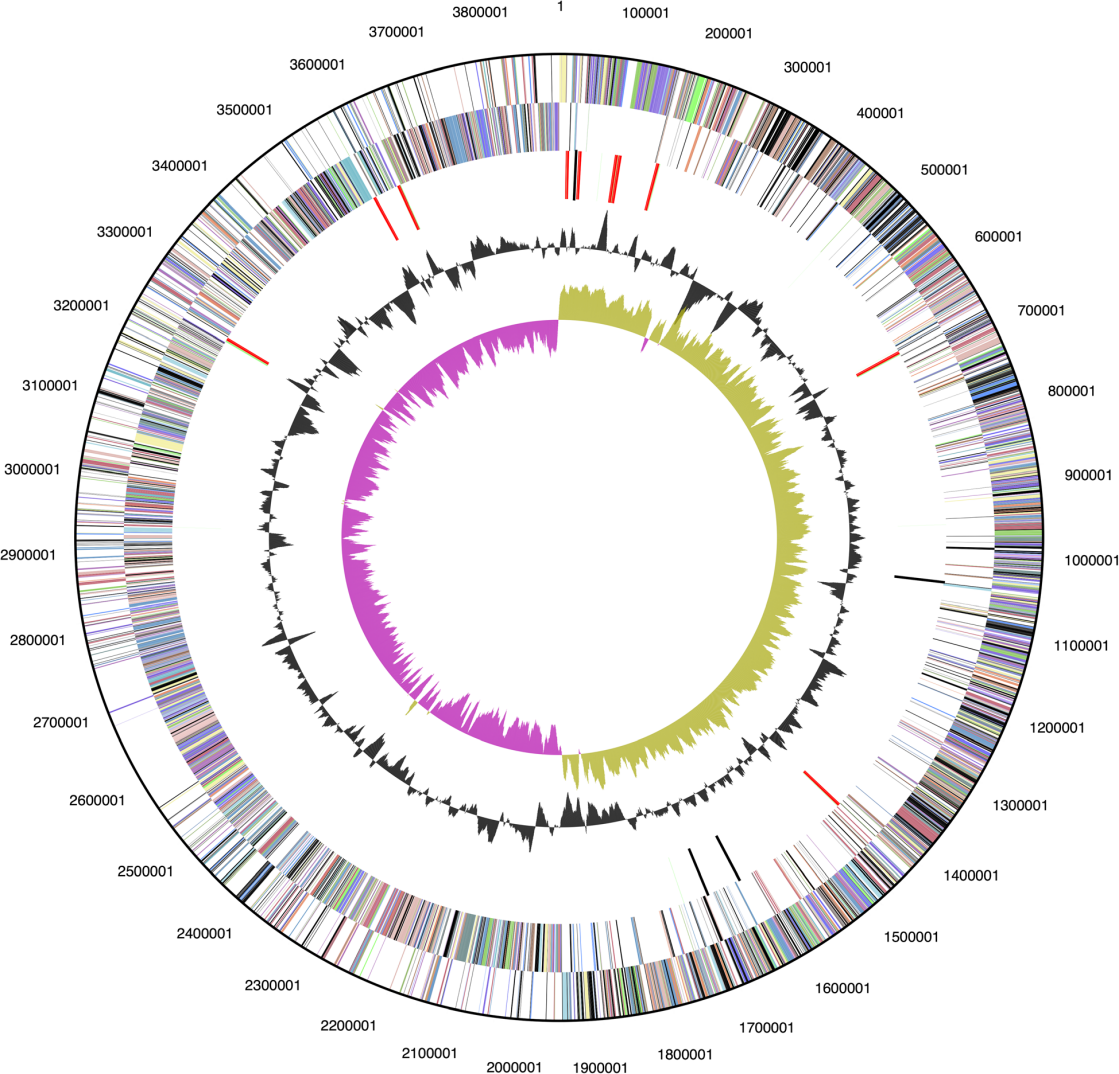
Graphical circular map of the chromosome. From outside to the center: Genes on forward strand (color by COG categories) Genes on reverse strand (color by COG categories) RNA genes (tRNAs green, rRNAs red, other RNAs black) GC content, GC skew

The genome of *G. thermoglucosidasius* C56-YS93 consists of one circular chromosome (Table 3 and Fig. 3) of 3,893,306 bp and two circular plasmids of 80,849 and 19,638 bp and an average G + C content of 43.93 % (Table 4). There are 90 tRNA genes, 27 rRNA genes and 4 “other” identified RNA genes. There are 4014 predicted protein-coding regions and 255 pseudogenes in the genome. A total of 2757 genes (66.7 %) have been assigned a predicted function while the rest have been designated as hypothetical proteins (Table 4). The numbers of genes assigned to each COG functional category are listed in Table 5. About 37 % of the annotated genes were not assigned to a COG or have an unknown function.

**Table 3:** Summary of genome: one chromosome and two plasmids

Label	Size (Mb)	Topology	INSDC identifier	RefSeq ID
Chromosome	3.65	Circular	CP002050.1	NC_14206.1
Plasmid 1	0.081	Circular	CP002836.1	NC_015665
Plasmid 2	0.020	Circular	CP002837.1	NC_015661

**Table 4:** Genome statistics

Attribute	Value	% of Total^a^
Genome size (bp)	3,993,793	100
DNA coding (bp)	3,437,131	86
DNA G+C (bp)	1,754,637	44
DNA Scaffolds	3	100
Total genes	4,135	100
Protein-coding genes	4,014	97
RNA genes	121	3
Pseudo genes	255	6
Genes in internal clusters	1,984	48
Genes with function prediction	1,257	30
Genes assigned to COGs	2,607	63
Genes with Pfam domains	3,278	79
Genes with signal peptides	161	4
Genes with transmembrane helices	948	23
CRISPR repeats	6	

^a^The total is based on either the size of the genome in base pairs or the total number of protein coding genes in the annotated genome

**Table 5:** Number of genes associated with general COG functional categories

Code	Value	Percent	Description
J	156	5.4	Translation, ribosomal structure and biogenesis
A	0	0	RNA processing and modification
K	195	6.8	Transcription
L	208	7.2	Replication, recombination and repair
B	1	0.03	Chromatin structure and dynamics
D	30	1.0	Cell cycle control, cell division, chromosome partitioning
V	44	1.5	Defense mechanisms
T	116	4.0	Signal transduction mechanisms
M	103	3.6	Cell wall/membrane/envelope biogenesis
N	61	2.1	Cell motility
U	48	1.7	Intracellular trafficking, secretion, and vesicular transport
O	100	3.5	Posttranslational modification, protein turnover, chaperones
C	207	7.2	Energy production and conversion
G	194	6.7	Carbohydrate transport and metabolism
E	285	9.9	Amino acid transport and metabolism
F	72	2.5	Nucleotide transport and metabolism
H	132	4.6	Coenzyme transport and metabolism
I	105	3.6	Lipid transport and metabolism
P	160	5.6	Inorganic ion transport and metabolism
Q	71	2.5	Secondary metabolites biosynthesis, transport and catabolism
R	330	11.5	General function prediction only
S	261	9.1	Function unknown
	1528	37.0	Not in COGs

The total is based on the total number of protein coding genes in the annotated genome

### Insights from the genome sequence

To better understand the unique features of *G. thermoglucosidasius* C56-YS93, whole genome comparisons were carried out between *G. thermoglucosidasius* C56-YS93 and *G. thermoglucosidasius* M10EXG (M10EXG) and *G. thermoglucosidasius* TNO-09.020 (TNO-09.020) (accession number: NZ_CM001483.1) using RAST genome annotation [37] and SEED curation into subsystems [38]. (The genome sequence of M10EXG is available from the Integrated Microbial Genomes (IMG) database [27, 39].) Basic features of the three genomes are shown in Table 6. Genome comparisons revealed that C56-YS93 possessed a number of unique features.

**Table 6:** *G. thermoglucosidasius* strains used in comparisons

*G. thermoglucosidasius*	C56-YS93	M10EXG	TNO-09.020
Chromosome size	3,993,793	3,773,252	3,740,238
Plasmids	2	0	0
Protein coding genes	4326	4228	4164
Isolation source	Hot Spring, YNP, United States	Sydney, New South Wales, Australia	Dairy production, Netherlands

### Xylan degradation cluster in G. thermoglucosidasius C56-YS93

The most significant unique feature of *G. thermoglucosidasius* C56-YS93 is a 26-gene cluster coding for xylan utilization not found in any *G. thermoglucosidasius* genome. Included in the cluster are regulatory elements, transporters, intracellular and extracellular xylanases, and enzymes involved in xylose metabolism (Table 7). Manual curation of the cluster indicates that the genes and organization of the *G. thermoglucosidasius* C56-YS93 xylan utilization cluster are essentially identical to those of the *G. stearothermophilus* cluster (Bst T-6) [40]. This identity suggests the cluster may be highly conserved within the xylanolytic geobacilli. No homologs of the corresponding *G. stearothermophilus* arabinan utilization [41] cluster genes are present in *G. thermoglucosidasius* C56-YS93, indicating *G. thermoglucosidasius* C56-YS93 is unable to utilize arabinan.

**Table 7:** Xylan degradation cluster of *Geobacillus thermoglucosidasius* C56-YS93

	Annotated protein function	C56-YS93	Bst T-6
1	Integral membrane sensor signal transduction histidine kinase	Geoth_2272	xynD
2	AraC family transcriptional regulator	Geoth_2271	xynC
3	Family 1 extracellular solute-binding protein	Geoth_2270	xynE
4	Binding-protein-dependent transporters inner membrane component	Geoth_2269	xynF
5	Binding-protein-dependent transporters inner membrane component	Geoth_2268	xynG
6	Aldose 1-epimerase	Geoth_2267	araK
7	Polysaccharide deacetylase	Geoth_2266	axe1
8	Xylan 1,4-beta-xylosidase	Geoth_2265	xynB2
9	Endo-1,4-beta-xylanase	Geoth_2264	xynA2
10	Family 1 extracellular solute-binding protein	Geoth_2262	aguE
11	Binding-protein-dependent transporters inner membrane component	Geoth_2261	aguF
12	Binding-protein-dependent transporters inner membrane component	Geoth_2260	aguG
13	Alpha-glucuronidase	Geoth_2259	aguA
14	Xylan 1,4-beta-xylosidase	Geoth_2258	xynB
15	PfkB domain-containing protein	Geoth_2257	kdgK
16	2-dehydro-3-deoxyphosphogluconate aldolase/4-hydroxy-2-oxoglutarate aldolase	Geoth_2256	kgdA
17	GntR family transcriptional regulator	Geoth_2255	uxuR
18	Uronate isomerase	Geoth_2254	uxaC
19	Mannonate dehydratase	Geoth_2253	uxuA
20	Short-chain dehydrogenase/reductase SDR	Geoth_2252	uxuB
21	Hypothetical protein	Geoth_2251	orfA
22	Endo-1,4-beta-xylanase	Geoth_2250	xynA1
23	Hypothetical protein	Geoth_2247	xynX
24	G-D-S-L family lipolytic protein	Geoth_2246	axe2
25	Xylose isomerase	Geoth_2243	xylA
26	Xylulokinase	Geoth_2242	xylB

### Nitrogen clusters in G. thermoglucosidasius C56-YS93

*G. thermoglucosidasius* C56-YS93 has a number of nitrogen utilization systems. The absence of an arabinan cluster in *G. thermoglucosidasius* C56-YS93 is the result of an 11-gene insert (Geoth_2276 through Geoth_2288) coding for a peptide utilization cluster that replaces part of the arabinan cluster. The cluster does not code for a secreted protease or peptidase, but contains an annotated five-gene ABC peptide transporter system and two intracellular peptidases. Downstream from the peptide utilization cluster is a 12-gene urea uptake and utilization cluster (Geoth_2301 through Geoth_2312). The organism contains clusters for reduction of nitrate to nitrite (Geoth_2197 through Geoth_2200) and reduction of nitrite to dintrogen (Geoth_3084 through Geoth_3090).

### Presence of plasmids in G. thermoglucosidasius C56-YS93

While the genomes of strains TNO-09.020 and M10EXG contain no plasmids, the genome of *G. thermoglucosidasius* C56-YS93 includes two plasmids, one of approximately 81 Kb and one of approximately 20 Kb. The 20 Kb plasmid contains genes coding for a number of small hypothetical proteins with no identifiable function. Among the annotated proteins, the 20Kb plasmid contains an annotated P4 phage/plasmid primase with no close homologs in other *Geobacillus* strains (Geoth_0020) and an annotated ArpU family phage transcriptional regulator (Geoth_0016). The 20 Kb plasmid contains an annotated transcriptional modulator of MazE/toxin, MazF (Geoth_0007) that may function in maintaining the plasmid. The 80 Kb plasmid contains a gene cluster that may function for proline and hydroxyproline capture, transport and metabolism. The cluster includes two peptidases (Geoth_3970 and Geoth_3979), a transport system and hydroxyglutarate oxidase cluster (Geoth_4004 and Geoth_3999), four annotated oxoprolinases (Geoth_3972, Geoth_3973, Geoth_3984, and Geoth_3987), and a hydantoin racemase (Geoth_3976) The plasmid also contains genes coding for proteins that metabolize proline to glutamate via proline dehydrogenase. (Geoth_3968 and Geoth_3969). BLAST analysis indicates that these two proteins are not common to *Geobacillus* species, but appear to have been acquired from an *Anoxybacillus* species. In addition, the 80 kb plasmid contains genes coding for eight proteins annotated as integrase or transposon-related and annotated death-on-curing and addiction module antidote proteins (Geoth_4023 and Geoth_4024) that may function in maintaining the plasmid.

### Prophage insert in G. thermoglucosidasius C56-YS93

**Fig. 4:**
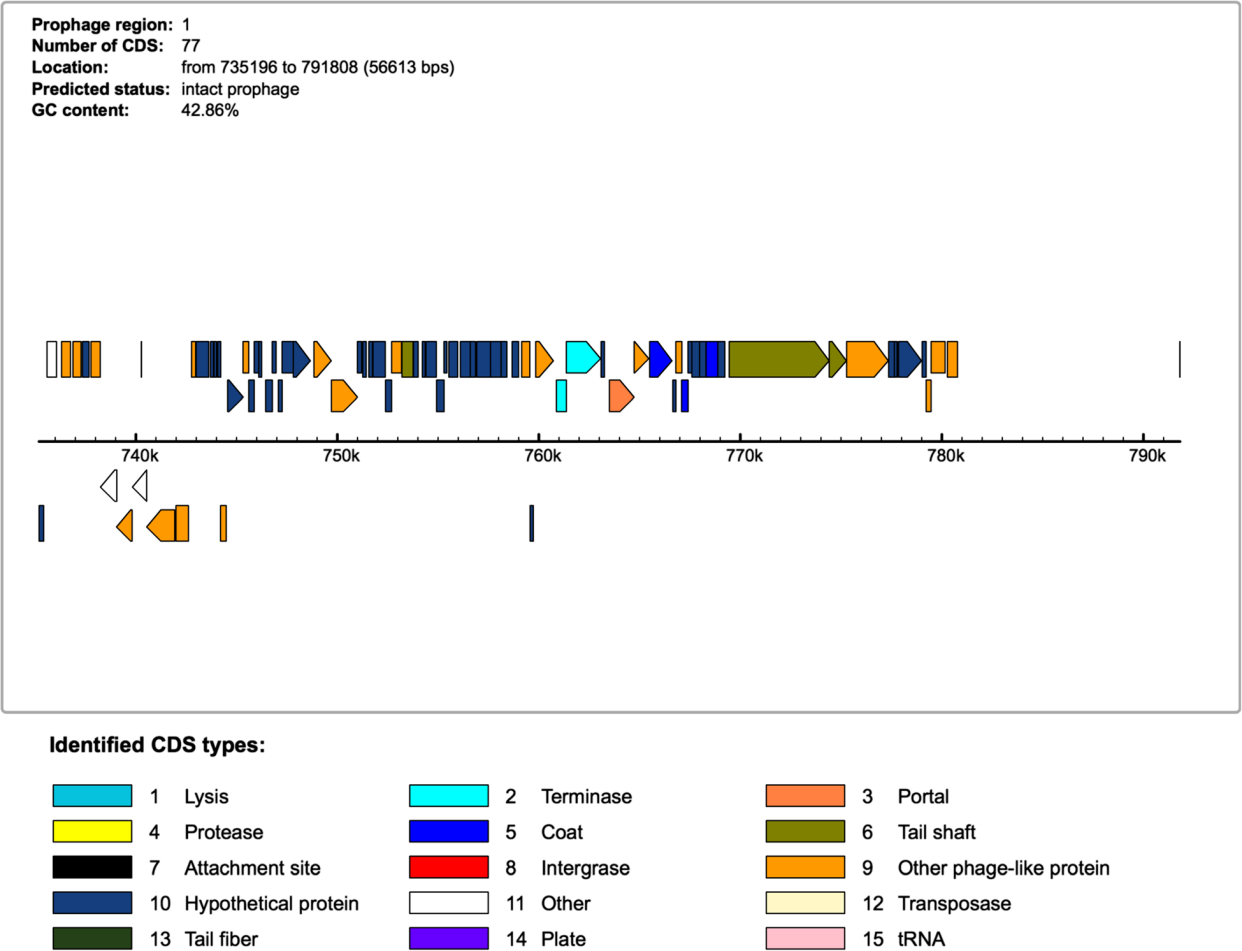
Prophage genes identified in *G. thermoglucosidasius* C56-YS93 using PHAST genome search software

Prophage analysis of the *G. thermoglucosidasius* C56-YS93 genome was performed using PHAST genome search software [42]. PHAST identified a 56 KB insert containing an intact prophage between 735,196 and 780,775 bp. The insert contains 75 genes, of which 51 are annotated as having a phage origin, 20 are annotated as hypothetical proteins and four are annotated as bacterial (Fig. 4). BLAST analysis indicates the phage proteins in the insert most closely match those of *Geobacillus* virus E2 (Accession: NC_009552.2) with 26 protein hits.

## Conclusions

*G. thermoglucosidasius* species were first isolated by Suzuki and given the name *Bacillus thermoglucosidasius* [43]. The organisms were reclassified as *Geobacillus* and their name corrected to *thermoglucosidasius* [9]. *G. thermoglucosidasius* C56-YS93 is the first *G. thermoglucosidasius* strain from a hot spring environment for which a whole genome sequence is available. While it is possible that *G. thermoglucosidasius* C56-YS93 was present only as wind-blown spores in the hot spring [18], there are a number of strong arguments for the growth of this and other *Geobacillus* species in hot springs. The first and most compelling argument is that, in our lab, boiled samples of Obsidian hot spring water resulted in isolation of no viable organisms, either in liquid culture or by plating. If *Geobacillus* spores were present in a significant quantity, a significant number of isolates would be expected. Secondly, we have been able to isolate *Geobacillus* species only from alkaline or neutral hot springs with temperatures between 60 and 80 °C, essentially the environment in which *Geobacillus* species can grow. No *Geobacillus* species were isolated from acidic hot springs located close to the alkaline and neutral springs. The isolation of wind-borne spore cultures would predict equal numbers of *Geobacillus* species isolated from acidic and alkaline springs. Thirdly, in our work, *Geobacillus* species and *Thermus* species were the predominant organisms isolated from Yellowstone hot springs under aerobic conditions. *Thermus* species share temperature and pH optima with *Geobacillus* species. *Thermus* species do not sporulate, so the presence of *Thermus* species cannot be attributed to wind-blown spores, but indicates the organism is growing in the hot spring. If these hot springs support growth of *Thermus* species, it would be difficult to argue that the hot springs can support growth of *Thermus* species but cannot support growth of with *Geobacillus* species. Finally, *Geobacillus* species have been isolated from microbial mats from other hot springs in Yellowstone [44].

*G. thermoglucosidasius**,* C56-YS93, appears to have a number of unique features as a result of its growth in the hot spring environment. The organism possesses a large xylan degradation cluster that increases the substrate range of this strain relative to the other two sequenced strains. A number of other biomass-degrading organisms have been identified in Obsidian Hot Spring [45], but this is the first reported biomass-degrading *Geobacillus* species from the hot spring. The organization of this cluster appears to match the glucuronic acid utilization cluster described for *G. stearothermophilus* [40], suggesting this cluster may be conserved in other *Geobacillus* species. *G. thermoglucosidasius* C56-YS93 possesses both chromosomal and plasmid-borne peptide utilization clusters that may allow the organism to scavenge proteins and peptides from the medium. *G. thermoglucosidasius* C56-YS93 also possesses the ability to reduce nitrate to dinitrogen, possibly utilizing nitrate as an alternate electron acceptor in the oxygen-poor high temperature environment. Genetic exchange with other *Geobacillus* species in the hot spring may be facilitated by the presence of the two plasmids not found in the other two strains. Further work is needed to identify the function of the genes present on these two plasmids and clarify the role they play in survival in the hot spring. Metagenomic analysis of samples from two other hot springs in Yellowstone National Park, Bear Paw and Octopus, shows the presence of active archaeal and bacterial phage populations [46, 47]. The prophage identified in *G. thermoglucosidasius* C56-YS93 (43.9 % G + C) is unrelated to the prophages identified in *Geobacillus* species Y412MC52 and Y412MC61 (52.3 % G + C), isolated from the same hot spring. This suggests that the identified prophage identified in *G. thermoglucosidasius* C56-YS93 may be specific to *G. thermoglucosidasius*, or to the lower G + C species. Additional work is needed to understand the relationship between *Geobacillus* species and the phages that infect them.

## Additional file

Additional file 1: Table S1. Associated MIGS record.

## Abbreviations

IMG: Integrate microbial genomes database
JGI: Joint Genome Institute
